# Suppression of NLRP3 Inflammasome by Erythropoietin via the EPOR/JAK2/STAT3 Pathway Contributes to Attenuation of Acute Lung Injury in Mice

**DOI:** 10.3389/fphar.2020.00306

**Published:** 2020-03-19

**Authors:** Fei Cao, Xinyi Tian, Zhongwang Li, Ya Lv, Jun Han, Rong Zhuang, Bihuan Cheng, Yuqiang Gong, Binyu Ying, Shengwei Jin, Ye Gao

**Affiliations:** Department of Anesthesia, Pain and Critical Care, The Second Affiliated Hospital and Yuying Children’s Hospital, Wenzhou Medical University, Wenzhou, China

**Keywords:** erythropoietin, acute lung injury, NLRP3 inflammasome, EPOR, JAK2/STAT3, NF-κB

## Abstract

Acute lung injury (ALI) and acute respiratory distress syndrome (ARDS) are common and devastating clinical disorders with high mortality and no specific therapy. An excessive inflammatory response results in the progression of ALI/ARDS, and the NLRP3 inflammasome is a key participant in inflammation. Erythropoietin (EPO), which is clinically used for anemia, reportedly exerts pleiotropic effects in ALI. However, whether EPO could protect against lipopolysaccharide (LPS)-induced ALI by regulating the NLRP3 inflammasome and its underlying mechanisms remain poorly elucidated. This study aimed to explore whether the therapeutic effects of EPO rely on the suppression of the NLRP3 inflammasome and the specific mechanisms in an LPS-induced ALI mouse model. ALI was induced in C57BL/6 mice by intraperitoneal (i.p.) injection of LPS (15 mg/kg). EPO was administered intraperitoneally at 5 U/g after LPS challenge. The mice were sacrificed 8 h later. Our findings indicated that application of EPO markedly diminished LPS-induced lung injury by restoring histopathological changes, lessened lung wet/dry (W/D) ratio, protein concentrations in bronchoalveolar lavage fluid (BALF) and myeloperoxidase (MPO) levels. Meanwhile, EPO evidently decreased interleukin-1β (IL-1β) and interleukin-18 (IL-18) secretion, the expression of NLRP3 inflammasome components including pro-IL-1β, NLRP3, and cleaved caspase-1 as well as phosphorylation of nuclear factor-κB (NF-κB) p65, which may be associated with activation of EPO receptor (EPOR), phosphorylation of Janus-tyrosine kinase 2 (JAK2) and signal transducer and activator of transcription 3 (STAT3). However, all the beneficial effects of EPO on ALI and modulation NLRP3 inflammasome were remarkably abrogated by the inhibition of EPOR/JAK2/STAT3 pathway and knockout (KO) of NLRP3 gene. Taken together, this study indicates that EPO can effectively attenuate LPS-induced lung injury in mice by suppressing the NLRP3 inflammasome, which is dependent upon activation of EPOR/JAK2/STAT3 signaling and inhibition of the NF-κB pathway.

## Introduction

Acute lung injury (ALI) and acute respiratory distress syndrome (ARDS) are common life-threatening critical illnesses with substantial morbidity and mortality (ARDS Definition Task Force et al., 2012; Rezoagli et al., 2017). Despite an improved understanding of the pathophysiology of these illnesses, there are still no effective pharmacologic therapies to treat patients with ALI/ARDS, and the hospital mortality is still as high as 46.1% (ARDS Definition Task Force et al., 2012). ALI/ARDS is characterized by exaggerated lung parenchyma inflammation, which leads to massive infiltration of activated neutrophils, progressive alveolar filling, and intractable hypoxemia (Giacomo Bellani et al., 2016). Hence, preventing exuberant inflammatory responses is suggested to be a potential strategy for the prevention and treatment of ALI.

The NLR family pyrin domain containing 3 (NLRP3) inflammasome is a multiprotein complex consisting of the sensor protein NLRP3, the adaptor protein ASC, and the effector protein caspase-1 (Lamkanfi and Dixit, 2014). NLRP3 is a cytoplasmic pattern recognition receptor (PRR) that can be activated by some pathogen-associated molecular patterns (PAMPs) or damage-associated molecular patterns (DAMPs), such as bacteria, viruses, and ATP (Swanson et al., 2019). Activation of NLRP3 leads to assembly of the adapter ASC, resulting in the autoactivation and cleavage of pro-caspase-1 into an enzymatically mature caspase-1, which further cleaves pro-IL-1β and pro-IL-18 into mature IL-1β and IL-18, respectively (Jo et al., 2016). Although activation of the NLRP3 inflammasome is a host defense mechanism to eliminate invading pathogens in infectious diseases, excessive activation of the NLRP3 inflammasome is also central to aberrant inflammation, progression to tissue injury, and organ dysfunction (Jorgensen and Miao, 2015). Recent studies have suggested that the NLRP3 inflammasome plays a pivotal role in the progression of ALI/ARDS caused by various pathogenic microorganisms, such as *influenza A virus* (Allen et al., 2009), *Pseudomonas aeruginosa* (Cohen and Prince, 2013), and *Staphylococcus aureus* (Miller et al., 2007).

Erythropoietin (EPO), an endogenous glycoprotein and a member of the type I cytokine superfamily, was first introduced as a hormone that promotes the proliferation, differentiation, and survival of erythroid progenitors by binding to its specific cell surface EPO receptor (EPOR) within the bone marrow (Fisher, 2003; Jelkmann, 2004). Accumulating evidence has demonstrated that EPO has cytoprotective effects in many pathological diseases, such as cardiac ischemia, kidney ischemia-reperfusion injury, and spinal cord injury (Yang et al., 2003; Lipsic et al., 2006; King et al., 2007). In addition, a number of studies have demonstrated the significant beneficial effects of EPO in various experimental models of ALI/ARDS, including experimental ALI/ARDS caused by acute necrotizing pancreatitis (Tascilar et al., 2007), ischemia-reperfusion (Wu et al., 2006, 2009), experimental sepsis induced by cecal ligation and puncture (Heitrich et al., 2016), LPS (Shang et al., 2009), and zymosan-induced non-septic shock (Cuzzocrea et al., 2006). The mechanisms by which EPO decreases the severity of lung injury are complicated and include protection of vascular integrity (Cuzzocrea et al., 2006), inhibition of the inflammatory response (Wu et al., 2009), and inhibition of free radical production and associated lipid peroxidation (Aoshiba et al., 2009). However, whether and how EPO can protect against LPS-induced ALI by regulating the NLRP3 inflammasome is still poorly elucidated.

In this study, we aimed to verify the role of EPO in modulating the NLRP3 inflammasome and consequently protecting against lung injury in LPS-induced experimental murine models of ALI. Additionally, our data clearly revealed that EPO could potently inhibit the NLRP3 inflammasome to mitigate lung injury, and this inhibition was potentially associated with the regulation of the EPOR/Janus-tyrosine kinase 2 (JAK2)/signal transducer and activator of transcription 3 (STAT3)/nuclear factor-κB (NF-κB) signaling axis. This study provides considerable evidence that EPO has a strong ability to effectively attenuate LPS-induced ALI in mice and might have therapeutic potential in the management of ARDS.

## Materials and Methods

### Materials

Recombinant human EPO (rhEPO) was purchased from Sunshine Pharmaceutical (Shenyang, China). Lipopolysaccharide (LPS from *Escherichia coli* 055:B5) was purchased from Sigma–Aldrich (St. Louis, MO, United States). EPO mimetic peptide 9 (EMP9, an EPOR antagonist) was synthesized by Fenghui Biotechnology (Changsha, China). Fedratinib (a JAK2 inhibitor), NSC 74859 (a STAT3 inhibitor), and BAY 11-7082 (an NF-κB inhibitor) were from MedChemExpress (Deer Park Dr, NJ, United States). The interleukin-1β (IL-1β), interleukin-18 (IL-18), myeloperoxidase (MPO) enzyme-linked immunosorbent assay (ELISA) kits, and anti-IL-1β antibody (catalog no. AF-401-NA) were from R&D Systems (Minneapolis, MN). Antibodies against phospho-NF-κB p65 (Ser536, catalog no. 3033S), NF-κB p65 (catalog no. 8242S), phospho-STAT3 (Tyr705, catalog no. 9145S), STAT3 (catalog no. 9139S), phospho-JAK2 (Tyr1007/1008), and JAK2 were obtained from Cell Signaling Technology (Beverly, MA, United States). Anti-Caspase-1 (catalog no. AG-20B-0042-C100), anti-NLRP3 (catalog no. AG-20B-0006-C100), and anti-ASC antibodies (catalog no. AG-25B-0006-C100) were from AdipoGen (San Diego, CA, United States).

### Animals and Experimental Protocol

Male specific-pathogen-free C57/BL6 mice, 8–10 weeks of age, were obtained from SLAC Animal Laboratory (Shanghai, China). NLRP3 knockout (KO) mice were obtained from the Experimental Animal Center of Zhejiang (Hangzhou, China). All mice were housed under controlled pathogen-free conditions in the Laboratory Animal Center of Wenzhou Medical University. All experiments were conducted in accordance with the Guide for the Care and Use of Laboratory Animals. This study was approved by the Animal Ethics Committees of the Wenzhou Medical University.

Wild-type (WT) mice were randomized into nine groups (*n* = 8): Control group, LPS group, LPS + EPO group, EPO group, LPS + EPO + EMP9 group, LPS + EPO + Fedratinib group, LPS + EPO + NSC 74859 group, LPS + EPO + BAY 11-7082 group, and LPS + EPO + DMSO group. The LPS-induced ARDS model was created by intraperitoneal (i.p.) injection of 15 mg/kg LPS in saline (or with an equal volume of saline as the Control group). EPO was administered 8 h (5 U/g, i.p.) after LPS or saline injection. The inhibitors EMP9 (1 mg/mouse, i.p.), Fedratinib (100 mg/kg, i.p.), NSC 74859 (5 mg/kg, i.p.), and BAY 11-7082 (20 mg/kg, i.p.) were injected 30 min before EPO administration (or an equal volume of DMSO was injected in the LPS + EPO + DMSO group). NLRP3 KO mice were randomized into three groups (*n* = 7): Control group, LPS group, and LPS + EPO group. The treatments were the same as those of WT mice.

### Histological Study

The left lung lobes were fixed in fresh 4% paraformaldehyde for 24 h, dehydrated and embedded in paraffin, and 5-μm-thick sections were made, which were finally stained with hematoxylin and eosin (H&E). The lung tissue sections were observed with a light microscope for lung histopathology. A semiquantitative scoring system was introduced to evaluate lung injury, which included alveolar congestion, alveolar hemorrhage, neutrophil infiltration, aggregation in the airspace or vessel wall, alveolar wall/hyaline membrane thickness, and inflammatory cell infiltration. The grading scale for the lung tissue pathologic findings was as follows: 0 = no injury; 1 = slight injury (25%); 2 = moderate injury (50%); 3 = severe injury (75%); and 4 = very severe injury (almost 100%). The lung injury score (total score: 0–16) was calculated as the sum of the scores, which has been described previously (Zhuo et al., 2018).

### Collection of Bronchoalveolar Lavage Fluid (BALF) and Total Protein Analysis

At the time of lavage, the mice were anesthetized with an i.p. injection of 1% pentobarbital sodium (50 mg/kg). The chest cavity was exposed, and then the trachea was intubated. PBS (1 mL) was slowly instilled into the lungs and withdrawn. The collected solutions were centrifuged at 1000 × *g* for 10 min at 4°C. The supernatant was harvested and stored at −80°C for further analysis. The total protein concentration of cell-free bronchoalveolar lavage fluid (BALF) was determined by the BCA method.

### ELISA

Mice were anesthetized with an i.p. injection of 1% pentobarbital sodium (50 mg/kg). Then, blood samples were collected from the eyeball after mice lost consciousness. After that, the blood samples were allowed to clot by leaving them undisturbed at room temperature for 30 min. Then, the clot was removed to obtain the serum by centrifugation at 1000 × *g* for 10 min at 4°C. Part of the right lung from each mouse was homogenized and centrifuged to obtain the lung tissue supernatants. The cytokines IL-1β and IL-18 in serum and IL-1β, IL-18, and MPO in lung tissue supernatants were quantified using murine ELISA kits according to the manufacturer’s recommendations.

### Lung Wet/Dry Ratio

When the lungs were isolated, the weights of the lungs were recorded as the wet weight, with the blood on the lungs blotted with filter paper. Then, the lungs were stored in an incubator at 60°C for 48 h. After that, the weights of the lungs were recorded as the dry weight. The lung wet/dry (W/D) ratio was used to evaluate the degree of pulmonary edema.

### Western Blotting

Lung tissue proteins were extracted using RIPA lysis buffer with PMSF and protein phosphatase inhibitor. The tissue homogenates were ultrasonicated three times for 3 s and then centrifuged at 12,000 × *g* for 30 min at 4°C. The total protein concentrations of the supernatants were determined with a BCA kit (Thermo Scientific, Rockford, IL, United States). Equal amounts of protein (30 μg) were separated on 10–12% SDS-PAGE gels and then transferred onto PVDF membranes. Membranes were blocked in 10% non-fat milk in TBST for 2 h and incubated overnight at 4°C with antibodies against the following antigens: NLRP3 (1:1000), Caspase-1 (1:1000), IL-1β (1:500), ASC (1:1000), p-JAK2 (1:1000), JAK2 (1:1000), p-STAT3 (1:1000), STAT3 (1:1000), NF-κB p-p65 (1:1000), and NF-κB p65 (1:1000). After three washes, the membranes were incubated with horseradish peroxidase-conjugated secondary antibodies (1:1000, Santa Cruz Company) at room temperature for 1 h. The protein bands were detected by ECL and visualized by a UVP gel imaging system (Upland, CA, United States). The band intensities were analyzed by using AlphaEaseFC (version 4.0).

### Quantitative Real-Time RT-PCR

Total RNA was isolated from lung tissues using TRIzol reagent(Invitrogen, Carlsbad, CA, United States) followed by phenol-chloroform extraction and ethanol precipitation (Fisher Scientific, Houston, TX, United States). Purified RNA concentrations were determined on a NanoDrop 1000 (Thermo Scientific). RNA samples were reverse transcribed into cDNA using an RT-PCR kit, according to the manufacturer’s instructions. Gene expression was assessed utilizing TaqMan Gene Expression Assays-On-Demand primer/probe sets and TaqMan Universal Master Mix (Life Technologies) on the Applied Biosystems 7500 Real-Time PCR system provided with SDS software. Twenty-microliter PCRs were performed using 2 μL of cDNA, 2 μL of primer/probe set, 10 μL of master mix, and 6 μL of DEPC-treated water by initially heating the samples to 50°C for 2 min and 95°C for 10 min, followed by 40 cycles of heating to 95°C for 15 s and 60°C for 1 min. Target gene expression levels were normalized to the levels of the housekeeping gene GAPDH, and the fold change was calculated using the 2^–△^^△^ Ct method.

The primers used for detection of the ASC gene were as follows:

Forward 5′-AGTCTGGAGCTGTGGCTACTGC-3′
Reverse 5′-TGAGTGCTTGCCTGTGTTGGTC-3′.

The primers used for detection of the caspase-1 gene were as follows:

Forward 5′-CGCATTTCCTGGACCGAGTGG-3′
Reverse 5′-GAGGGCAAGACGTGTACGAGTG-3′.

The primers used for detection of the IL-18 gene were as follows:

Forward 5′-CGACCGAACAGCCAACGAAT-3′
Reverse 5′-GGGTCACAGCCAGTCCTCTT-3′.

The primers used for detection of the IL-1β gene were as follows:

Forward 5′-ACAGCAGCATCTCGACAAGAGC-3′
Reverse 5′-CCACGGGCAAGACATAGGTAGC-3′.

The primers used for detection of the NLRP3 gene were as follows:

Forward 5′-CCTGGTCTGCTGGATTGTGTGC-3′
Reverse 5′-AGTCGTGGTCTTGGAGGTCTGG-3′.

### Statistical Analysis

The normal distribution data are expressed as the mean ± SD and were analyzed by one-way ANOVA or two-way ANOVA followed by Tukey’s *post hoc* test for multiple comparisons. The non-normal distribution data are reported as medians and ranges (5th–95th percentile). Kruskal–Wallis test followed by Dunn multiple comparisons *post hoc* test was performed for data of non-normal distribution. Significance was determined at the *p* < 0.05 level. Statistical analyses were performed using Prism 7.0 software (GraphPad Software, San Diego, CA, United States).

## Results

### EPO Mitigated LPS-Induced Acute Lung Injury in Mice

**FIGURE 1:**
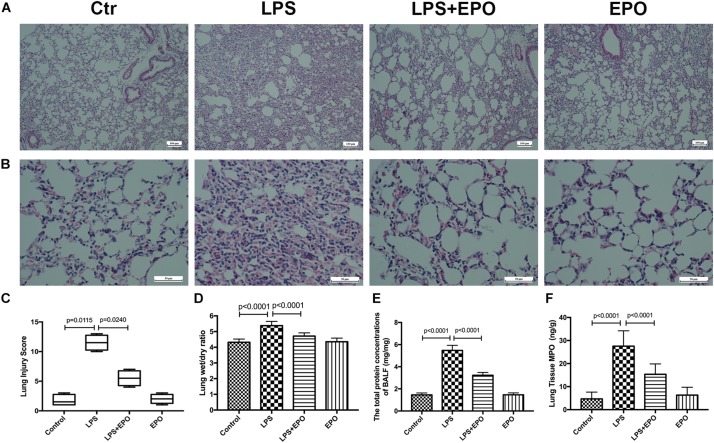
EPO mitigated LPS-induced acute lung injury in mice. EPO (5 U/g) was injected into the peritoneal cavity of mice after LPS (15 mg/kg) stimulation. The mice were sacrificed 8 h later, and the effects of EPO were assessed in **(A,B)** hematoxylin and eosin-stained sections (original magnification × 100, × 400). Lung injury scores **(C)** were recorded from 0 (no damage) to 16 (maximum damage) according to the criteria described in Section “Materials and Methods.” The lung wet/dry ratio **(D)** was tested to evaluate pulmonary edema. The total protein concentrations in the BALF **(E)** were measured to reflect the integrity of the pulmonary alveolar-capillary barrier. Myeloperoxidase (MPO) concentrations in lung tissues **(F)** were measured by ELISA to quantitatively determine the resolution of infiltrated cells. The lung injury score data are presented as medians and ranges (5th –95th percentile), and the differences among groups were assessed by Kruskal–Wallis test and the *post hoc* test (Dunn’s method) was applied to investigate the differences one by one. *n* = 4 per group. The other data are presented as the mean ± SD. The differences of **D**–**F** were assessed by one-way ANOVA and the *post hoc* test (Turkey method) was applied to investigate the differences one by one. *n* = 8 per group.

The morphologic changes of lung tissues were assessed by using H&E staining. In contrast to the normal pulmonary histology in the Control group and EPO group, LPS exposure dramatically induced aggravated pathologic changes with severe hemorrhage, interstitial edema, and marked inflammatory cell infiltration; however, these changes were effectively ameliorated by EPO administration (Figures 1A,B). The quantification of ALI scores was also in parallel with the pathophysiological changes (Figure 1C). Moreover, the pulmonary edema and microvascular permeability were estimated by the W/D weight ratio and protein leakage in BALF, respectively. The W/D ratio and total protein concentration in BALF were much higher in the LPS group than in the Control group, whereas EPO treatment significantly reduced pulmonary edema and microvascular permeability (Figures 1D,E). In addition, lung MPO expression, reflecting neutrophil infiltration, was markedly decreased in the LPS + EPO group compared with the LPS group (Figure 1F).

### EPO Downregulated the Expression and Concentration of IL-1β and IL-18 in LPS-Induced Acute Lung Injury

**FIGURE 2:**
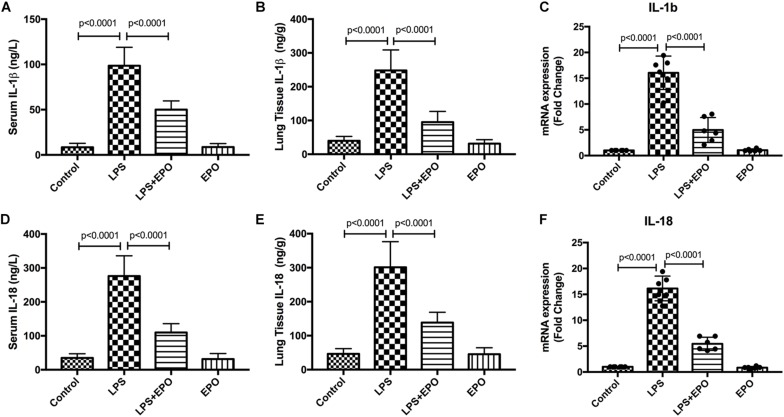
EPO downregulated the expression and concentration of IL-1β and IL-18 in LPS-induced ALI. EPO (5 U/g) was injected into the peritoneal cavity of mice after LPS (15 mg/kg) stimulation. The mice were sacrificed 8 h later, and the serum was obtained to measure the concentrations of IL-1β and IL-18 **(A,D)** by ELISA. The lung tissues were harvested to measure the concentrations of IL-1β and IL-18 **(B,E)** by ELISA, and the mRNA expression levels of pro-IL-1β and pro-IL-18 **(C,F)** were measured by QPCR. The data are presented as the mean ± SD. The differences among groups were assessed by one-way ANOVA and the *post hoc* test (Turkey method) was applied to investigate the differences one by one. *n* = 8 per group.

Because the inflammatory response is involved in the ALI process, we explored the effect of EPO on inflammatory mediators. The concentrations of IL-1β and IL-18 in serum and lung tissue homogenates were dramatically increased in LPS-induced ALI relative to the control, whereas these cytokines were markedly downregulated by EPO treatment (Figures 2A,B,D,E). In addition, the mRNA expression levels of IL-1β and IL-18 were also in parallel with these changes (Figures 2C,F).

### EPO Suppressed the NLRP3 Inflammasome in LPS-Induced Acute Lung Injury

**FIGURE 3:**
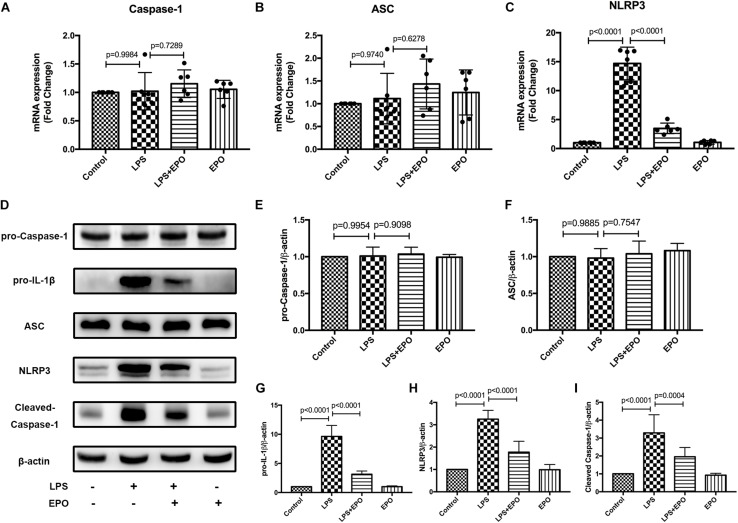
EPO suppressed the NLRP3 inflammasome in LPS-induced ALI. EPO (5 U/g) was injected into the peritoneal cavity of mice after LPS (15 mg/kg) stimulation. The mice were sacrificed 8 h later, and the lung tissues were obtained to measure the mRNA expression levels of caspase-1, ASC, and NLRP3 **(A–C)** by QPCR. In addition, the protein expression levels of pro-caspase-1, pro-IL-1β, ASC, NLRP3, and cleaved caspase-1 **(D–I)** were detected by Western blotting. The data are presented as the mean ± SD. The differences among groups were assessed by one-way ANOVA and the *post hoc* test (Turkey method) was applied to investigate the differences one by one. *n* = 8 per group.

The NLRP3 inflammasome plays a vital role in the generation of IL-1β and IL-18, and we examined the mRNA and protein expression levels of NLRP3 inflammasome-associated proteins in lung tissues. The mRNA levels of NLRP3 and the protein expression levels of pro-IL-1β, NLRP3, and cleaved caspase-1 were markedly increased in LPS-induced ALI compared with the Control group, whereas these levels were distinctly decreased by EPO treatment (Figures 3C,D,G–I). However, there were no changes in pro-caspase-1 and ASC in any of the groups (Figures 3A,B,E,F). These results implied that EPO suppressed the NLRP3 inflammasome by inhibiting NLRP3 protein expression and then reducing cleaved caspase-1, ultimately decreasing the levels of the mature cytokines IL-1β and IL-18.

### EPO Activated the EPOR/JAK2/STAT3 Pathway and Inhibited NF-κB Activation in LPS-Induced Acute Lung Injury

**FIGURE 4:**
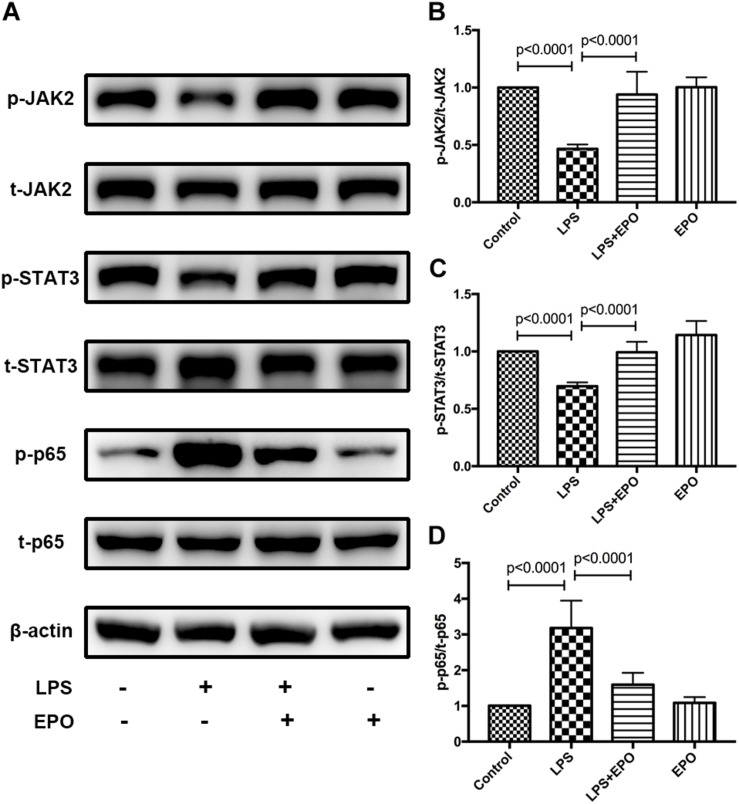
EPO activated the EPOR/JAK2/STAT3 pathway and inhibited NF-κB activation in LPS-induced ALI. EPO (5 U/g) was injected into the peritoneal cavity of mice after LPS (15 mg/kg) stimulation, and the mice were sacrificed 8 h later. The lung tissues were harvested to measure the protein expression levels of p-JAK2, t-JAK2, p-STAT3, t-STAT3, NF-κB p-p65, and NF-κB t-p65 **(A–D)** by Western blotting. The data are presented as the mean ± SD. The differences among groups were assessed by one-way ANOVA and the *post hoc* test (Turkey method) was applied to investigate the differences one by one. *n* = 8 per group.

Next, we explored the possible underlying mechanism involved in the regulatory effect of EPO on ALI. The activities of JAK2, STAT3, and NF-κB, which are important intracellular signaling pathways induced by EPO (Kuhrt and Wojchowski, 2015; Ma et al., 2016; McGraw and List, 2017), were measured by Western blotting in lung tissue homogenates. We discovered that LPS challenge clearly reduced the expression of p-JAK2 and p-STAT3 and upregulated the activation of NF-κB p-p65, while these effects were obviously reversed by EPO treatment (Figures 4A–D).

### The Effects of EPO on Acute Lung Injury Were EPOR/JAK2/STAT3/NF-κB Signal Axis Dependent

**FIGURE 5:**
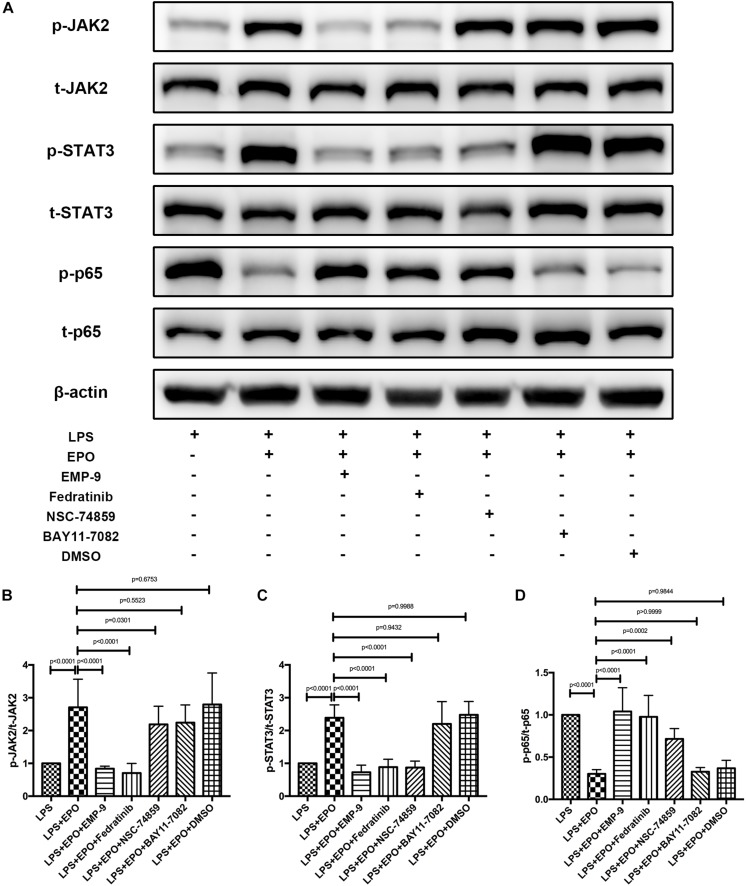
The protective effects of EPO on acute lung injury were EPOR/JAK2/STAT3/NF-κB signaling axis dependent. EMP9 (an EPOR antagonist, 1 mg/mouse), Fedratinib (a JAK2 inhibitor, 100 mg/kg), NSC 74859 (a STAT3 inhibitor, 5 mg/kg), BAY 11-7082 (an NF-κB inhibitor, 20 mg/kg), and DMSO (solvent of inhibitors) were injected into the peritoneal cavity 30 min before EPO treatment. Then, EPO (5 U/g) was injected into the peritoneal cavity of mice after LPS (15 mg/kg) stimulation, and the mice were sacrificed 8 h later. The lung tissues were harvested to measure the protein expression of p-JAK2, t-JAK2, p-STAT3, t-STAT3, NF-κB p-p65, and NF-κB t-p65 **(A–D)** by Western blotting. The data are presented as the mean ± SD. The differences among groups were assessed by one-way ANOVA and the *post hoc* test (Turkey method) was applied to investigate the differences one by one. *n* = 8 per group.

Signaling is activated when EPO binds the extracellular domains of its receptor, the EPOR, which is the first step in the EPO-mediated signaling pathway (McGraw and List, 2017). These results are also supported by the finding that the EPOR antagonist EMP9 markedly abolished the EPO-induced increase in p-JAK2 and p-STAT3 as well as the reduction of NF-κB p-p65 (Figures 5A–D). To further corroborate the correlations among JAK2, STAT3, and NF-κB, we found that the JAK2 inhibitor Fedratinib also markedly reversed the expression of p-JAK2, p-STAT3, and NF-κB p-p65 induced by EPO stimulation, while inhibitors of STAT3 (NSC 74859) and NF-κB (BAY 11-7082) had no effect on the activation of p-JAK2. These data indicated that JAK2 is upstream of the STAT3 and NF-κB signaling pathways. Additionally, neither p-JAK2 nor p-STAT3 activation was abrogated by the NF-κB inhibitor BAY 11-7082, while the STAT3 inhibitor significantly affected the expression of NF-κB p-p65 (Figures 5A–D). Together, these data demonstrated that EPO plays a role in regulating the EPOR/JAK2/STAT3/NF-κB signaling axis in ALI.

### The Inhibitors of the EPOR/JAK2/STAT3 Pathway Reversed the Suppressing Effect of EPO on the NLRP3 Inflammasome in LPS-Induced Acute Lung Injury

**FIGURE 6:**
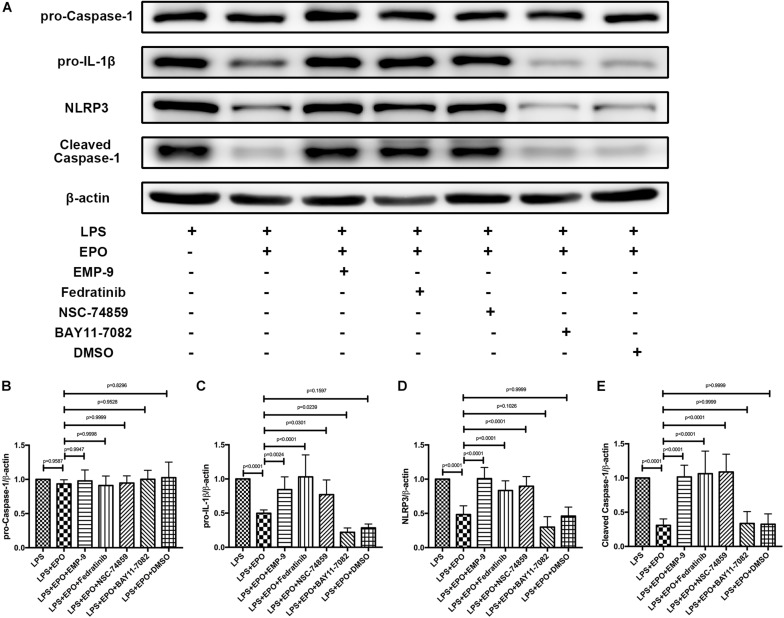
Inhibitors of the EPOR/JAK2/STAT3 pathway reversed the suppressing effect of EPO on the NLRP3 inflammasome. EMP9 (an EPOR antagonist, 1 mg/mouse), Fedratinib (a JAK2 inhibitor, 100 mg/kg), NSC 74859 (a STAT3 inhibitor, 5 mg/kg), BAY 11-7082 (an NF-κB inhibitor, 20 mg/kg), and DMSO (solvent of the inhibitors) were injected into the peritoneal cavity 30 min before EPO treatment. Then, EPO (5 U/g) was injected into the peritoneal cavity of mice after LPS (15 mg/kg) stimulation. The mice were sacrificed 8 h later, and the lung tissues were harvested to measure the protein expression levels of pro-caspase-1, pro-IL-1β, NLRP3, cleaved caspase-1 **(A–E)** by Western blotting. The data are presented as the mean ± SD. The differences among groups were assessed by one-way ANOVA and the *post hoc* test (Turkey method) was applied to investigate the differences one by one. *n* = 8 per group.

Considering that EPO could interact with EPOR and activate the JAK2/STAT3 axis to inhibit the NF-κB signaling pathway, our further work aimed to explore whether EPO-mediated downregulation of the NLRP3 inflammasome is associated with this pathway. As illustrated in Figures 6A,C–E, the specific inhibitors of EPOR (EMP9), JAK2 (Fedratinib), and STAT3 (NSC 74859) significantly abolished the EPO-induced reduction in pro-IL-1β and NLRP3 components (NLRP3, cleaved caspase-1), whereas the inhibition of NF-κB by BAY 11-7082 did not change the suppressive effect of EPO on NLRP3 inflammasome. Notably, there were no differences in pro-caspase-1 among any of the groups (Figures 6A,B), which is consistent with the previous results.

### EPO Protected Against Lung Injury Through the EPOR/JAK2/STAT3/NF-κB Signaling Pathway

**FIGURE 7:**
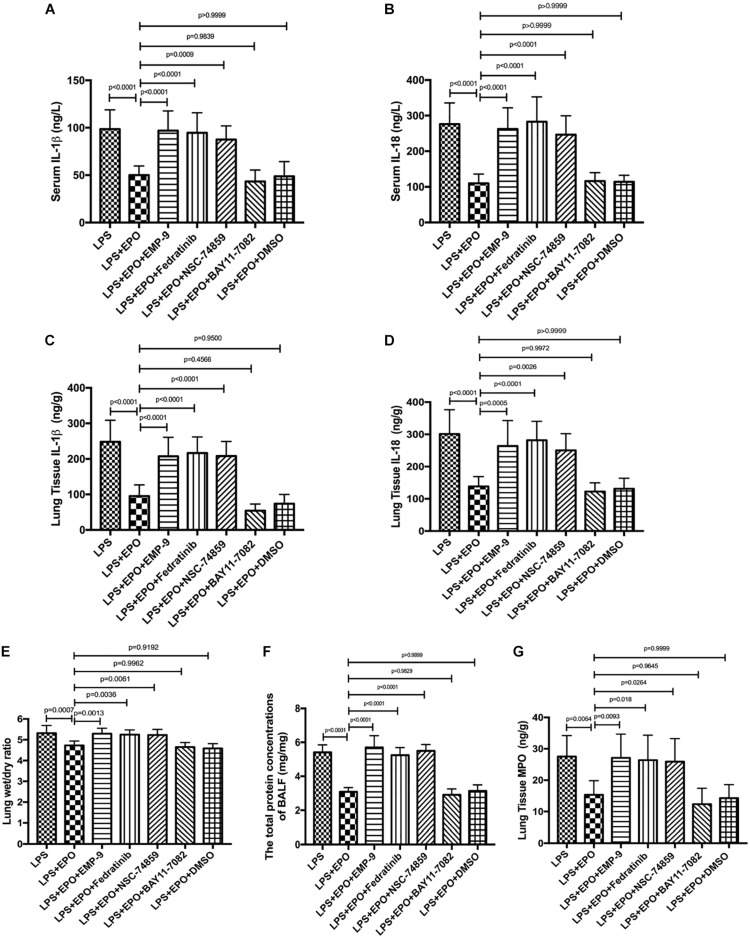
EPO protected against lung injury through the EPOR/JAK2/STAT3 and NF-κB signaling pathways. EMP9 (an EPOR antagonist, 1 mg/mouse), Fedratinib (a JAK2 inhibitor, 100 mg/kg), NSC 74859 (a STAT3 inhibitor, 5 mg/kg), BAY 11-7082 (an NF-κB inhibitor, 20 mg/kg), and DMSO (solvent of the inhibitors) were injected into the peritoneal cavity 30 min before EPO treatment. Then, EPO (5 U/g) was injected into the peritoneal cavity of mice after LPS (15 mg/kg) stimulation. The mice were sacrificed 8 h later, and the sera were obtained to measure the concentrations of IL-1β and IL-18 **(A,B)** by ELISA. The BALF was obtained to measure the total protein concentrations **(F)**. In addition, the lung tissues were harvested to measure the lung wet/dry ratio **(E)**, and the concentrations of IL-1β, IL-18, and MPO **(C,D,G)** were measured by ELISA. The data are presented as the mean ± SD. The differences among groups were assessed by one-way ANOVA and the *post hoc* test (Turkey method) was applied to investigate the differences one by one. *n* = 8 per group.

We further verified the contribution of EPOR/JAK2/STAT3 and NF-κB to EPO-induced lung protection by using specific inhibitors. The results revealed that the EPOR antagonist (EMP9), JAK2 inhibitor (Fedratinib), and STAT3 inhibitor (NSC 74859) abolished EPO-mediated alleviation of lung injury with higher IL-1β and IL-18 concentrations, W/D ratio, BALF protein levels, and MPO production (Figures 7A–G). Considering that the similar tendency between LPS + EPO group and LPS + EPO + BAY 117082 group and BAY 117082 was given 30 min prior to EPO administration, the inhibitory effect of EPO and BAY 117082 were suggested to be upon suppressing the same pathway. Thus, the protective roles of EPO in ALI were dependent on the upregulation of the EPOR/JAK2/STAT3 signaling axis and the inhibition of the NF-κB pathway.

### The Protective Effects of EPO Against Acute Lung Injury Are Dependent on the Inhibition of NLRP3

**FIGURE 8:**
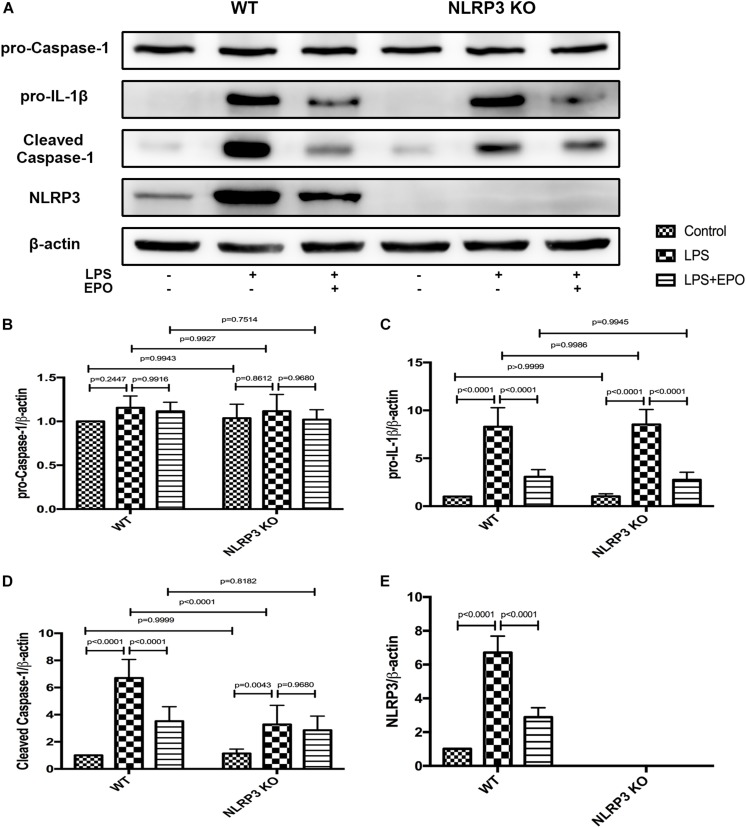
The knockout of NLRP3 mimicked the effects of EPO on the NLRP3 inflammasome in ALI. EPO (5 U/g) was injected into the peritoneal cavity of mice after LPS (15 mg/kg) stimulation of NLRP3 knockout (KO) mice and wild-type (WT) mice, and the mice were sacrificed 8 h later. The lung tissues were harvested to measure the protein expression levels of pro-caspase-1, pro-IL-1β, cleaved caspase-1, and NLRP3 **(A–E)** by Western blotting. The data are presented as the mean ± SD. The differences among groups were assessed by two-way ANOVA and the *post hoc* test (Turkey method) was applied to investigate the differences one by one. *n* = 7 per group.

To further ascertain the role of NLRP3 in the regulation of the NLRP3 inflammasome by EPO, we compared the inflammasome-associated proteins pro-caspase-1, pro-IL-1β, NLRP3, and cleaved caspase-1 between WT and NLRP3 KO mice. Regardless of the stimulus, the expression of pro-caspase-1 displayed no significant difference between WT and NLRP3 KO mice (Figures 8A,B). Figures 8A,E evidently confirm the absolute deletion of NLRP3 in NLRP3 KO mice. LPS could increase the expression of pro-IL-1β, and EPO could also decrease the expression of pro-IL-1β in NLRP3 KO mice. Thus, the deletion of NLRP3 had no effect on the regulation of pro-IL-1β (Figures 8A,C). Additionally, EPO administration significantly reversed the increase in cleaved caspase-1 induced by LPS in WT mice, whereas there was no significant change in its production between the LPS + EPO and LPS groups in NLRP3 KO mice (Figures 8A,D). These data suggested that EPO inhibits NLRP3-dependent Caspase-1 activation, but not NLRP3-independent activation.

**FIGURE 9:**
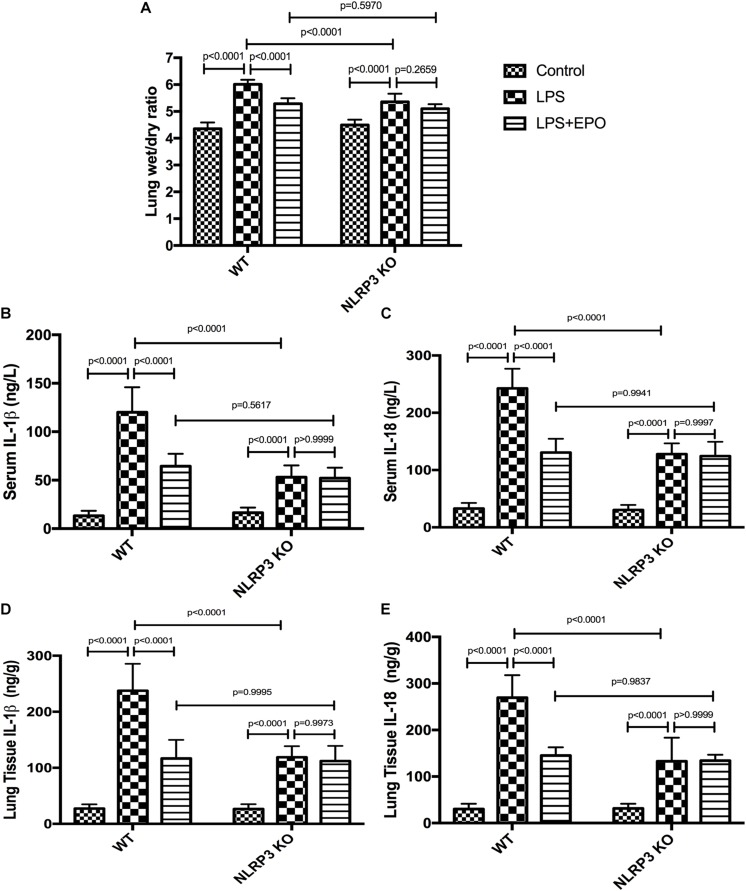
EPO attenuated lung injury and inhibited IL-1β and IL-18 secretion, which was dependent on NLRP3 in LPS-induced ALI. EPO (5 U/g) was injected into the peritoneal cavity of mice after LPS (15 mg/kg) stimulation in NLRP3 knockout (KO) mice and wild-type (WT) mice, and the mice were sacrificed 8 h later. The lung tissues were harvested to measure the lung wet/dry ratio **(A)** and the concentrations of IL-1β and IL-18 **(D,E)** by ELISA. The sera were obtained to measure the concentrations of IL-1β and IL-18 **(B,C)** by ELISA. The data are presented as the mean ± SD. The differences among groups were assessed by two-way ANOVA and the *post hoc* test (Turkey method) was applied to investigate the differences one by one. *n* = 7 per group.

Given that NLRP3 is an important component of the NLRP3 inflammasome, we sought to determine whether NLRP3 mediates EPO-induced lung protection. The inhibitory effects of EPO on the W/D lung ratio, IL-1β, and IL-18 in both serum and lung tissue were abolished in NLRP3 KO mice (Figures 9A–E).

## Discussion

In our mouse model of LPS-induced ALI, EPO administration potently repaired the morphological and histological changes in lung tissue, as well as reduced lung W/D ratio, total protein concentration in the BALF, and MPO concentration. Moreover, EPO markedly alleviated NLRP3 inflammasome activation with decreased NLRP3 and pro-IL-1β mRNA and protein expression and reduced IL-1β and IL-18 production, and upregulation of JAK2 and STAT3 phosphorylation and inhibition of NF-κB p65 phosphorylation were involved in these processes. However, the inhibition of EPOR, JAK2, and STAT3 and deletion of NLRP3 not only abolished suppression of the NLRP3 inflammasome but also diminished the beneficial effects of EPO on ALI. These results suggest that EPO can attenuate lung inflammation of LPS-induced ALI by suppressing the NLRP3 inflammasome in a process that depends on the EPOR/JAK2/STAT3/NF-κB signaling axis.

Uncontrolled inflammatory responses are thought to play essential roles in the pathogenesis of ALI, which results in extensive neutrophil infiltration, massive release of inflammatory mediators, a widespread increase in capillary permeability and severe interstitial edema (Han and Mallampalli, 2015). Therefore, effective control of inflammation appears to be a critical step in the treatment of ALI. An elevated lung W/D ratio and protein concentration in the BALF account for enhanced capillary permeability and increased pulmonary edema in LPS-induced ALI (Bhattacharya and Matthay, 2013), which are pronouncedly reversed by EPO treatment. Furthermore, EPO alleviated LPS-initiated MPO production, an indirect measure of neutrophil sequestration in the tissue. All these data are consistent with the histopathological study indicating that EPO administration markedly mitigated the inflammatory injury induced by LPS challenge.

NLRP3 inflammasome activation is a tightly regulated process, which is considered to be a two-step process, with the first a priming step and the second an activation step (Mangan et al., 2018). Priming includes at least two aspects of regulation. The first aspect is the upregulation of pro-IL-1β and the inflammasome sensor NLRP3 at the transcriptional level. This transcriptional upregulation can be stimulated through the recognition of diverse PAMPs or DAMPs, which is mediated by PRRs and is followed by activation of the NF-κB pathway to induce associated gene transcription (Bauernfeind et al., 2009; Dinarello, 2009; Franchi et al., 2009). Thus, the activation of the inflammasome requires NF-κB signaling pathway activation as its first signal. In addition, the second aspect of priming is the rapid posttranslational modifications (PTMs) of the inflammasome component NLRP3, which stabilize NLRP3 in an autosuppressed inactive state (Filardy et al., 2016; Barry et al., 2018; Shim and Lee, 2018; Lopez-Castejon, 2019). After priming, the second activation step occurs. Various NLRP3 activators can induce full activation of the NLRP3 inflammasome by initiating the formation of a multiprotein complex, which consists of NLRP3, ASC, and pro-caspase-1 (Petrilli et al., 2007; Zhou et al., 2011; Triantafilou et al., 2013; Kelley et al., 2019).

In this study, we found that EPO markedly reduced the concentrations of IL-1β and IL-18 in serum and lung tissue homogenates, implying a suppressive effect of EPO on the NLRP3 inflammasome. To explore whether EPO influenced the first step of NLRP3 inflammasome activation, we examined the mRNA and protein expression of NLRP3 inflammasome components in lung tissue. We found that EPO significantly downregulated the mRNA and protein expression of pro-IL-1β, NLRP3, and cleaved caspase-1. These results suggested that EPO suppressed the NLRP3 inflammasome mainly by inhibiting the priming step of NLRP3 inflammasome activation. However, the mRNA and protein expression levels of pro-caspase-1 and ASC were not different among the groups. These results suggest that transcriptional regulation of the inflammasome components ASC and pro-Caspase-1 is not required for inflammasome activation, as these proteins are sufficient in the steady state. These findings are consistent with previous studies (Bauernfeind et al., 2009; Franchi et al., 2009).

Recently, accumulating studies have proven that EPO exerts its biological functions through targeting cell surface receptor EPOR (Farrell and Lee, 2004; Kuhrt and Wojchowski, 2015), which is extensively distributed in numerous tissues and organs. In lung tissue, EPOR has been identified as being expressed on mesothelium, chondrocytes, smooth muscle fibers, alveolar macrophages, neutrophils, vascular endothelial cells, bronchial and alveolar epithelium (MacRedmond et al., 2009; Yasuda et al., 2010). Additionally, the EPO/EPOR signaling pathway has also been reported to exhibit pleiotropic cytoprotection in ALI, including alleviation of endothelial cells proliferation (Beleslin-Čokić et al., 2011), inhibition of epithelial cells apoptosis, and promotion of pulmonary angiogenesis (Heitrich et al., 2016). Once EPO binds to EPOR, JAK2 is activated through phosphorylation (designated as p-JAK2). Subsequently, this event further results in the phosphorylation and dimerization of STATs, which are direct substrates of JAK (Digicaylioglu and Lipton, 2001; Kuhrt and Wojchowski, 2015). Our present study illustrated that EPO facilitated the phosphorylation of JAK2 and STAT3 in LPS-induced ALI. Phosphorylated STAT3 inhibited NF-κB p65 phosphorylation and nuclear translocation, resulting in the suppression of NLRP3, pro-IL-1β, and pro-IL-18 expression. Consistent with our studies, Zhu et al. (2010) reported that EPO bound to the EPOR and subsequently activated JAK2/STAT3 signaling to reduce neuronal apoptosis and induce angiogenesis.

Given the crucial role of the NLRP3 inflammasome and the participants of EPOR/JAK2/STAT3 in EPO-mediated actions, we used specific inhibitors and NLRP3 KO mice to further clarify the underlying mechanisms. The inhibition of EPOR, JAK2, and STAT3 abrogated the effect of EPO on the mitigation of lung injury as well as the suppression of the NLRP3 inflammasome. Moreover, the downregulation of cleaved caspase-1, IL-1β, and IL-18 and the decrease in the lung W/D ratio caused by EPO were abolished when the NLRP3 gene was knocked out. These data suggest that EPO suppressed the NLRP3 inflammasome in a manner dependent on the EPOR/JAK2/STAT3/NF-κB pathway.

Our study provided evidence that NLRP3 inflammasome implicated in development of ALI and EPO potently protected against lung injury via suppressing the first signal of NLRP3 inflammasome priming. As activation of NLRP3 inflammasome requires the priming step and the activation step, whether EPO can regulate the second signal of NLRP3 activation is required for further exploration.

## Conclusion

**FIGURE 10:**
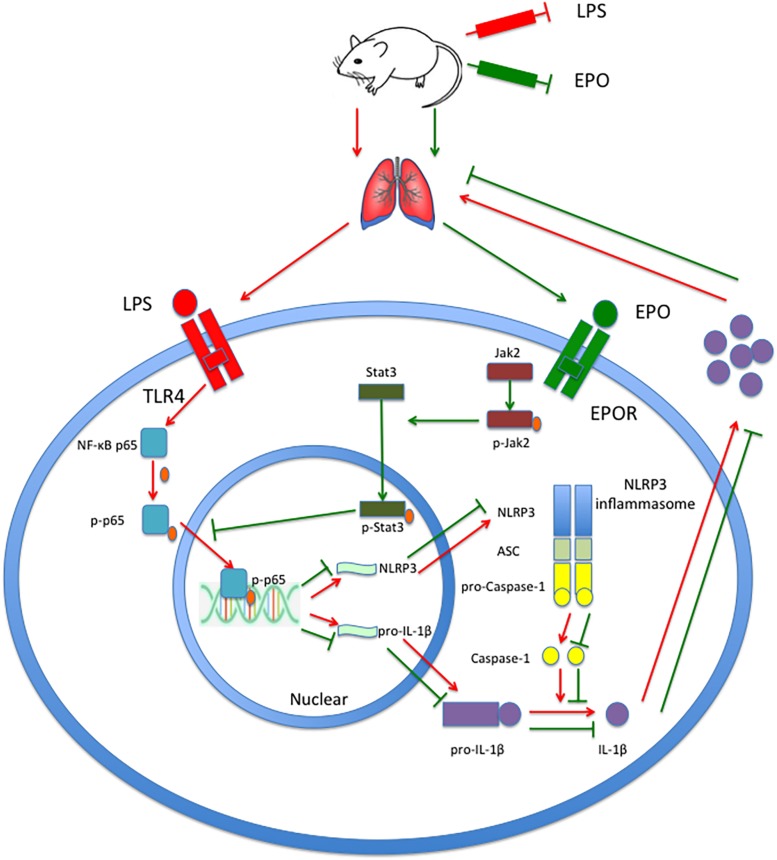
EPO attenuated LPS-induced acute lung injury by suppressing the NLRP3 inflammasome via the EPOR/JAK2/STAT3/NF-κB pathway in mice. EPO binds to EPOR, further phosphorylates JAK2 to p-JAK2, and controls the kinase activity of JAK2 to phosphorylate STAT3 to p-STAT3. Subsequently, p-STAT3 inhibits the phosphorylation of NF-κB p65 and the transcriptional activity of NF-κB p-p65 to attenuate the transcription and expression of NLRP3 and pro-IL-1β, which consequently leads to a decrease in mature IL-1β levels and the mitigation of lung injury.

In summary, this study demonstrates that EPO can attenuate the ALI induced by LPS in mice. Moreover, EPO markedly suppresses the NLRP3 inflammasome by decreasing NLRP3 and pro-IL-1β mRNA and protein expression and reducing IL-1β and IL-18 production. Upregulation of JAK2 and STAT3 phosphorylation and inhibition of NF-κB p65 phosphorylation are involved in these processes. Moreover, the inhibition of EPOR, JAK2, and STAT3 and the deletion of NLRP3 not only abolished the suppression of the NLRP3 inflammasome but also diminished the beneficial effects of EPO on ALI. Taken together, these findings indicate that the EPOR/JAK2/STAT3/NF-κB pathway mediates the NLRP3 inflammasome suppression and lung protection induced by EPO in LPS-induced ALI. As shown in Figure 10, our findings improve the understanding of the anti-inflammatory effect mediated by EPO via suppression of NLRP3 inflammasome activation and elucidate the underlying mechanisms. Considering the strong inflammatory potential of NLRP3 inflammasome, as well as its central role in inflammatory diseases, EPO might have therapeutic potential in the management of numerous inflammatory diseases.

## Data Availability Statement

The datasets used and/or analyzed during the current study are available from the corresponding author on reasonable request.

## Ethics Statement

The animal study was reviewed and approved by Animal Ethics Committees of the Wenzhou Medical University. All experiments were performed in accordance with the National Institutes of Health guide for the care and use of laboratory animal.

## Author Contributions

SJ and YeG conceived the study. FC performed the experiments, analyzed the data, and wrote the manuscript. XT, YL, and JH helped with the experimental work. ZL helped with the data analysis. RZ, BC, YuG, and BY discussed experimental designs and revised the manuscript. All authors discussed the data, commented on the manuscript, and read and approved the final manuscript.

## Conflict of Interest

The authors declare that the research was conducted in the absence of any commercial or financial relationships that could be construed as a potential conflict of interest.
